# Immunohistochemical Expression of p16, p53, BCL2, and Cyclin D1 in Gastrointestinal Stromal Tumors: Correlation With Clinicopathological Parameters

**DOI:** 10.7759/cureus.93731

**Published:** 2025-10-02

**Authors:** Marwa M Zaki, Eman T Enan, Heba Hany

**Affiliations:** 1 Pathology Department, Faculty of Medicine, Mansoura University, Mansoura, EGY

**Keywords:** bcl2, cyclin d1, gastrointestinal stromal tumors, p16, p53

## Abstract

Introduction

Gastrointestinal stromal tumors (GISTs) represent the predominant mesenchymal neoplasms within the gastrointestinal tract. Although some clinicopathological features can help estimate the risk of tumor progression, there is still a need for more reliable immunohistochemical markers to predict the tumor’s behavior. The current study aims to evaluate the immunohistochemical expression of p16, p53, BCL2, and Cyclin D1 in GISTs and to analyze their correlations with the clinicopathological characteristics and survival outcomes of the studied cases.

Methods

This retrospective cohort study included 65 cases of GISTs, retrieved from the archive of the Pathology Laboratory at the Gastroenterology Center, Mansoura University, between 2014 and 2018. Patients’ clinical and pathological data were revised. Three tissue microarray blocks were constructed. Immunohistochemical staining for p16, p53, BCL2, and Cyclin D1 was performed. Clinicopathological correlation and survival analysis were analyzed using appropriate statistical methods.

Results

In this study, p16, p53, BCL2, and Cyclin D1 expressions were observed in 15 cases (23.1%), 21 cases (32.3%), 52 cases (80%), and 57 cases (87.7%), respectively. Regarding p16 expression, it was significantly associated with tumor site, risk category, mitotic index, local recurrence, distant metastasis, lymph node involvement, and the expression of CD117, BCL2, p53, and Cyclin D1. p16 expression was also identified as an independent predictor of local recurrence and distant metastasis. Concerning p53 expression, it was significantly correlated with tumor size, site, risk category, mitotic index, local recurrence, distant metastasis, and the expression of p16, BCL2, and Cyclin D1. In contrast, BCL2 expression showed no significant association with most clinicopathological parameters, except for distant metastasis. However, it was significantly associated with the expression of p16, p53, and Cyclin D1. With respect to Cyclin D1 expression, it was significantly associated with tumor site, local recurrence, lymph node involvement, distant metastasis, and the expression of p16, p53, and BCL2. Regarding survival analysis, positive expression of p16 and p53 was significantly associated with shorter disease-free and overall survival. High BCL2 expression was correlated with reduced overall survival, whereas high Cyclin D1 expression showed a non-significant trend toward improved survival.

Conclusion

Our findings suggest that p16 and p53 expressions are valuable prognostic markers in GISTs, significantly associated with aggressive behavior and poor outcomes. p16 may serve as an independent predictor of recurrence and metastasis, while p53 could aid in risk stratification. Although BCL2 and Cyclin D1 showed associations with tumor aggressiveness, their prognostic roles remain uncertain. Further multicenter, standardized studies are needed to validate the clinical relevance of these markers.

## Introduction

Gastrointestinal stromal tumors (GISTs) represent the predominant mesenchymal neoplasms within the gastrointestinal tract. Their precursors are the interstitial cells of Cajal, which are located between the circular and longitudinal layers of the muscularis propria. GISTs are uncommon tumors, making up only about 1% to 2% of all malignant neoplasms in the gastrointestinal system [1,2]. Most tumors affect the stomach and small intestine. GISTs mainly affect adults aged 60 to 70 years, with no gender predominance [3,4].

The behavior of GISTs varies from incidental small tumors to those with widespread metastasis. The primary treatment for GISTs is total surgical excision. Post-surgery adjuvant therapy, especially with imatinib or other tyrosine kinase inhibitors (TKIs), relies on GIST risk classifications, which remain inadequate and debatable [4].

The 2018 Version 2 National Comprehensive Cancer Network (NCCN) guidelines, the most recent European Society for Medical Oncology (ESMO)/European Reference Network on Rare Adult Solid Cancers (EURACAN) guidelines, and the French Intergroup Clinical Practice guidelines identified key risk factors: mitotic rate, tumor size, and tumor site. Moreover, tumor rupture, cellular atypia, and necrosis were acknowledged as independent risk factors. Meanwhile, other prognostic indicators, such as different gene mutations, have also been linked to patient outcomes [3,5].

Mutations in the KIT proto-oncogene, which encodes the receptor tyrosine kinase c-KIT, along with subsequent discoveries of platelet-derived growth factor receptor alpha (PDGFRA) mutations, led to the development of small-molecule targeted therapies using TKIs in the treatment of GISTs. Nevertheless, these mutations alone are insufficient to drive the progression of GISTs from low to high risk. Further chromosomal alterations and resulting disruptions in cell cycle regulation are crucial for advancing tumor development [2].

The tumor suppressor protein cyclin-dependent kinase inhibitor 2A, p16INK4a (p16), inhibits the cell cycle by arresting cells in G1 before S phase entry. Loss of p16 protein has been identified as a predictor of a poor clinical outcome in a variety of human tumors and has also been reported with high-risk GISTs [5,6].

The tumor protein p53 (TP53) gene encodes the tumor suppressor protein p53, often referred to as the guardian of the genome, and is mutated in most human malignancies, albeit with varying frequencies across different tumor types. TP53 mutations are generally infrequent (<5%) in GIST cohorts but have been linked to high-risk disease [2].

B-cell lymphoma 2 (BCL2) is an essential membrane protein mainly present on the outer mitochondrial membrane. The overexpression of BCL2 may provide a selective survival advantage to neoplastic cells through its anti-apoptotic effects. The overexpression of BCL2 in GISTs may result from the intrinsic activation of KIT. Furthermore, its recurrent overexpression may be targeted for adjuvant or alternative treatment [7].

Cyclin D1 is activated during the G1 phase of the cell cycle, subsequently activating cyclin-dependent kinase 4 (CDK4) and cyclin/CDK complexes, facilitating the progression to the S phase of the cell cycle [8]. Cyclin D1 was shown to be overexpressed in KIT-independent GISTs, indicating its function in tumor proliferation and resistance when KIT signaling is lost [9]. Therefore, the inhibition of Cyclin D1 may induce anti-proliferative effects and promote apoptosis [10].

The current study aims to evaluate the immunohistochemical expression of p16, p53, BCL2, and Cyclin D1 in GISTs and to analyze their correlations with the clinicopathological characteristics and survival outcomes of the studied cases.

## Materials and methods

Study design

This retrospective cohort study included 65 cases of GISTs retrieved from the archive of the Surgical Pathology Laboratory at Mansoura University's Gastroenterology Center between 2014 and 2018. The inclusion criteria were the availability of comprehensive clinical data, medical reports, and paraffin blocks. Cases lacking comprehensive clinical data or available paraffin tissue blocks were excluded from the study.

Clinical characteristics and histopathological assessment

The pathology reports of the included cases, along with hematoxylin and eosin (H&E) and immunohistochemical stained slides for cluster of differentiation 117 (CD117), discovered on GIST-1 (DOG1), smooth muscle actin (SMA), cluster of differentiation 34 (CD34), and S100 protein (S100), were examined and revised to validate the diagnosis and evaluate the clinicopathological characteristics, including age, gender, tumor location and size, surgical margins, lymph node involvement, local recurrence, and distant metastasis. Both disease-free survival (DFS) and overall survival (OS) were calculated for all patients and subsequently analyzed to assess their prognostic implications.

All cases were re-evaluated, and risk stratification was conducted in accordance with the latest criteria recommended by the NCCN guidelines and the WHO Classification of Digestive System Tumors (fifth edition, 2019). Mitotic count was assessed by counting mitoses in the region with the highest mitotic activity and reported as the number of mitotic figures per 5 mm², following the updated guidelines, which replaced the previous method based on 50 high-power fields, to ensure standardization and reproducibility [11].

Tissue microarray (TMA) construction

After examination of H&E-stained slides, a representative cellular-qualified area of the tumor was marked for each case. The corresponding paraffin block was retrieved from our pathology archive. Six 0.6 mm thick cores were extracted from each case and placed into the recipient blocks. Three TMA blocks were constructed using a Beecher manual micro-arrayer, following a specific design map for each block.

Immunohistochemical staining procedure

Four-micrometer-thick tissue sections were obtained from previously constructed TMA blocks. Sections underwent deparaffinization, rehydration, and antigen retrieval using the BT-Link system (Dako, Glostrup, Denmark), following the manufacturer’s instructions. Immunostaining was performed using the automated Dako autostainer Link 48 platform. The following primary antibodies were used: Rabbit monoclonal anti-human p16 antibody (clone QR019; Quartett, Berlin, Germany), ready-to-use, Mouse monoclonal anti-human p53 protein (clone DO-7; Dako, Glostrup, Denmark), ready-to-use, Mouse monoclonal anti-human BCL2 oncoprotein (clone 124; Dako, Glostrup, Denmark), ready-to-use, Rabbit monoclonal anti-human Cyclin D1 (clone EP12; Dako, Glostrup, Denmark), ready-to-use.

Detection was performed using the Dako EnVision FLEX system with High pH buffer, according to the manufacturer’s protocol. 3,3′-Diaminobenzidine (DAB) was utilized as the chromogen, while hematoxylin was used for counterstaining.

Appropriate positive controls were included for each marker. Negative controls were conducted concurrently by omitting the primary antibody to verify staining specificity.

Immunohistochemical analysis

p16 expression was evaluated based on the percentage of immunoreactive tumor cells, defined by strong cytoplasmic staining with or without concurrent nuclear staining. A threshold of 10% was applied as the cutoff point, with cases categorized as negative (0%-9%) or positive (≥10%). Similarly, p53 expression was considered positive when ≥10% of tumor cells exhibited strong nuclear staining.

BCL2 and Cyclin D1 expression were assessed using a semi-quantitative scoring system that accounted for both staining intensity and the percentage of positively stained cells. For Cyclin D1, only nuclear staining was evaluated, whereas cytoplasmic staining was considered for BCL2. Staining intensity was graded on a scale from 0 (no staining) to 3 (strong staining), and the proportion of positive cells was scored as follows: 0 (no staining), 1 (1-10%), 2 (11-50%), 3 (51-80%), and 4 (>80%). The final score was calculated by multiplying the intensity and percentage scores, yielding a composite score ranging from 0 to 12. Tumors were subsequently categorized into three groups based on this score: negative (0), low expression (1-5), and high expression (6-12).

Statistical analysis

The data were analyzed using SPSS software (IBM SPSS Statistics for Windows, IBM Corp., Version 25, Armonk, NY). Qualitative data were expressed numerically and as percentages. Quantitative data were presented as mean ± standard deviation. The significance of the acquired results was determined at the 0.05 level. Chi-square and Monte Carlo tests were used to compare qualitative data across groups as needed. Non-normally distributed data were compared using the Kruskal-Wallis test and the Mann-Whitney U test. The Student's t-test was used to compare two independent groups with non-normally distributed data. A one-way ANOVA test was used to examine numerous independent groups, using a post hoc Tukey test for pairwise comparisons. Spearman's rank-order correlation measures the strength and direction of a linear link between two non-normally distributed continuous or ordinal variables. Using the enter approach, binary logistic regression is used to analyze the influence of multiple independent predictors on a binary outcome. Survival analysis was performed using the Kaplan-Meier method, with differences between groups assessed by the log-rank test. Median survival times were estimated along with their corresponding 95% confidence intervals (CIs).

## Results

The clinicopathological characteristics of the studied cohort are summarized in Table 1. The patients’ mean age was 60.7 years, while the mean tumor size was 8 cm. The stomach was the predominant primary site (40 cases, 61.5%), followed by the small intestine (12 cases, 18.5%). Regarding risk stratification, 30 cases (46.2%) were classified as very low/low risk, seven cases (10.8%) as intermediate risk, and 28 cases (43.1%) as high risk. Most tumors had free surgical margins (60 cases, 92.3%). Local recurrence was observed in 11 cases (16.9%), distant metastasis in 16 cases (24.6%), and lymph node involvement in seven cases (10.8%). Immunohistochemically, CD117 was expressed in 44 cases (67.7%), and DOG1 in 34 cases (52.3%). In contrast, CD34, SMA, and S100 showed lower expression rates.

**Table 1:** Clinicopathological features of the included cases. CD34: cluster of differentiation 34; CD117: cluster of differentiation 117; DOG1: discovered on GIST-1; S100: S100 protein; SMA: smooth muscle actin

Clinicopathological features		Study group
		(N = 65)
Age (years)	Mean age	60.72 ± 12.68
	Age range	(23-83)
Tumor size (cm)	Mean size	8
	Size range	(1-35)
Tumor site	Stomach	40 (61.5%)
	Small intestine	12 (18.5%)
	Rectum	9 (13.8%)
	Pelvi-abdominal	1 (1.5%)
	Pancreas	1 (1.5%)
	Mesentery	2 (3.1%)
Mitotic index	≤ 5 per 5 mm^2^	38 (58.5%)
	> 5 per 5 mm^2^	27 (41.5%)
Risk stratification	Very low/low	30 (46.2%)
	Intermediate	7 (10.8%)
	High	28 (43.1%)
Surgical cut margins	Free	60 (92.3%)
	Infiltrative	5 (7.7%)
Local recurrence	Absent	54 (83.1%)
	Present	11 (16.9%)
Metastasis	Absent	49 (75.4%)
	Present	16 (24.6%)
Lymph nodes status	Negative	58 (89.2%)
	Positive	7 (10.8%)
CD117 expression	Negative	21 (32.3%)
	Positive	44 (67.7%)
DOG1 expression	Negative	31 (47.7%)
	Positive	34 (52.3%)
CD34 expression	Negative	40 (61.5%)
	Positive	25 (38.5%)
SMA expression	Negative	50 (76.9%)
	Positive	15 (23.1%)
S100 expression	Negative	55 (84.6%)
	Positive	10 (15.4%)

**Figure 1:**
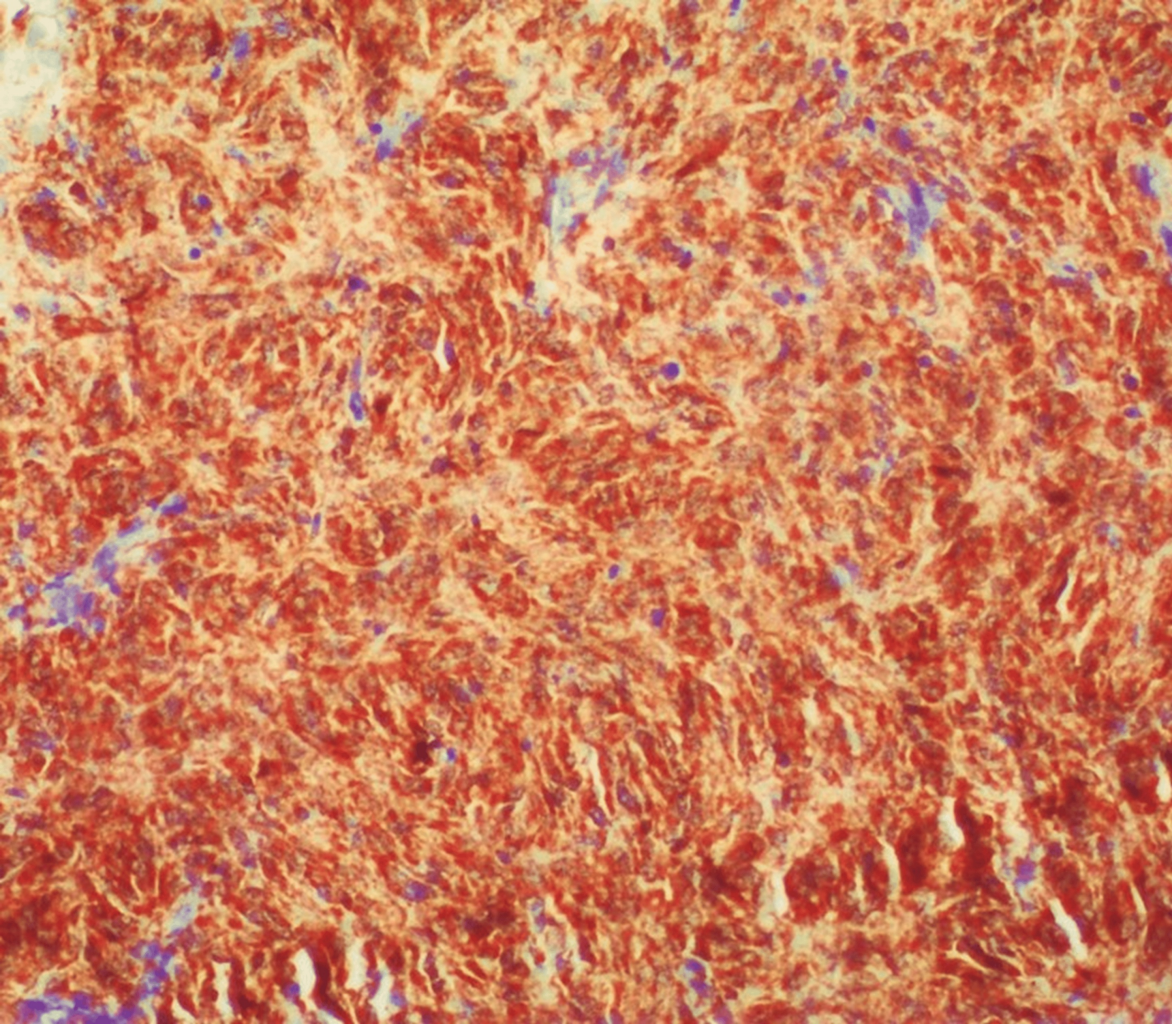
Strong cytoplasmic p16 expression in a gastrointestinal stromal tumor (GIST) from small intestine with high-risk stratification (p16, ×200).

**Figure 2:**
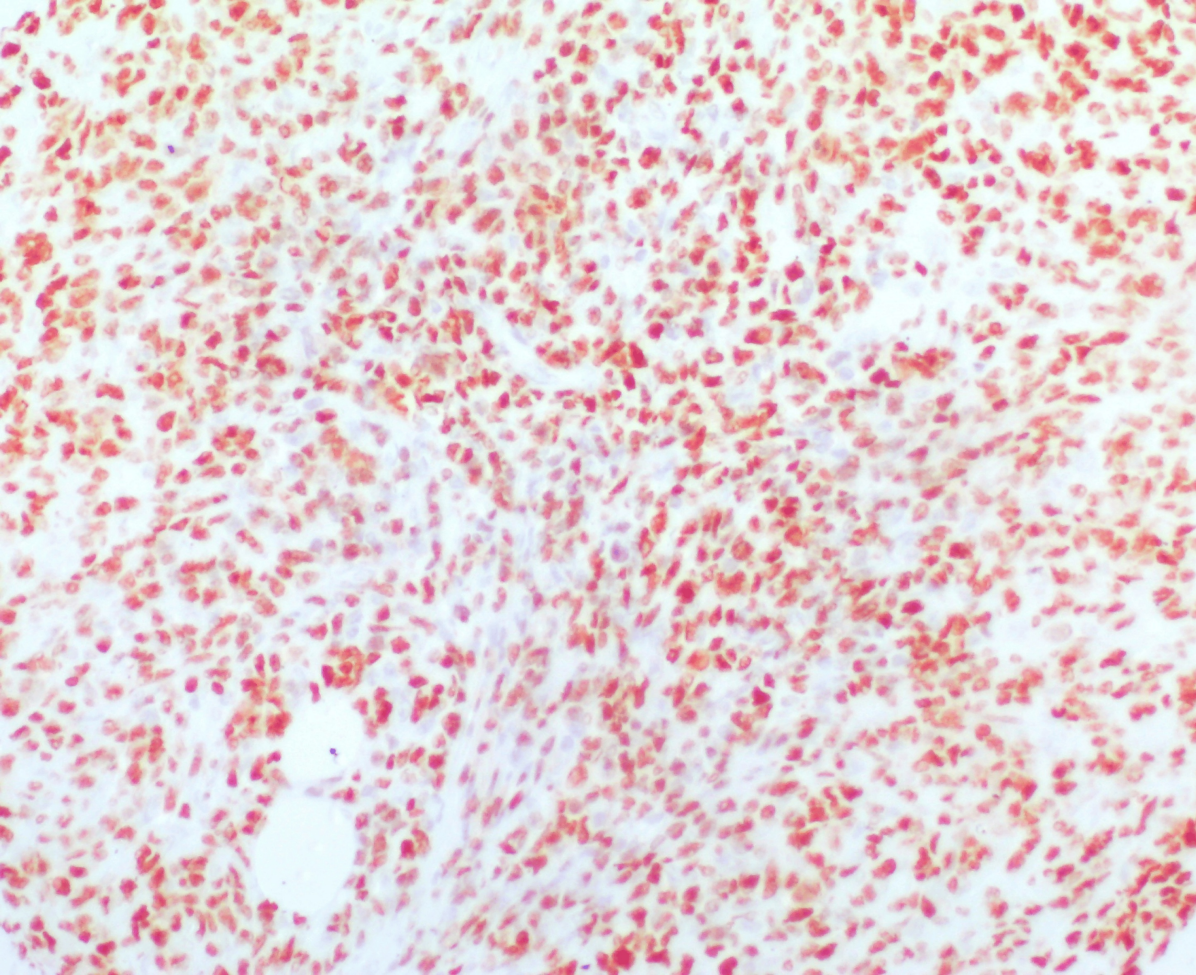
Strong nuclear p53 expression in a gastric gastrointestinal stromal tumor (GIST) with high risk stratification (p53, ×200).

**Figure 3:**
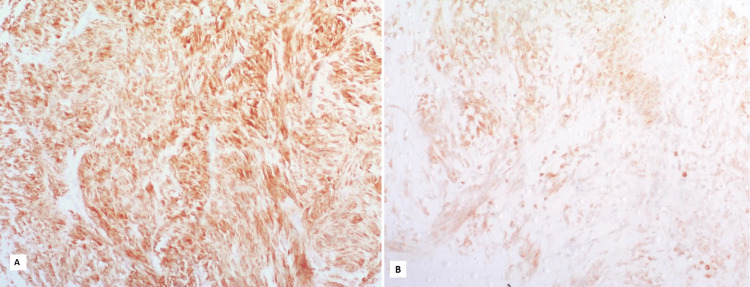
High cytoplasmic BCL2 expression in a gastrointestinal stromal tumor (GIST) from the small intestine with high-risk stratification. B: Low cytoplasmic BCL2 expression in a GIST from the small intestine with high risk stratification (BCL2, ×200).

**Figure 4:**
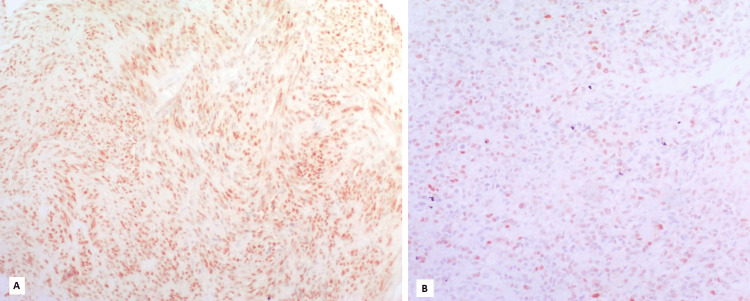
High nuclear Cyclin D1 expression in a gastric gastrointestinal stromal tumor (GIST) with intermediate risk stratification. B: Low nuclear Cyclin D1 expression in a gastric GIST with high risk stratification.

Regarding the immunohistochemical expression of p16, p53, BCL2, and Cyclin D1. We reported that 15 cases (23.1%) were positive for p16, 21 cases (32.3%) were positive for p53, 52 cases (80%) were positive for BCL2, and 57 cases (87.7%) were positive for Cyclin D1 (Table 2, Figures 1-4).

**Table 2:** Immunohistochemical expression of p16, p53, BCL2, and Cyclin D1 in the studied cases. BCL2: B-cell lymphoma 2; p16: cyclin-dependent kinase inhibitor 2A, p16INK4a; p53: tumor protein p53.

IHC marker			Number of cases
			N = 65
p16 expression	Negative		50 (76.9%)
	Positive		15 (23.1%)
p53 expression	Negative		44 (67.7%)
	Positive		21 (32.3%)
BCL2 expression	Negative		13 (20%)
	Positive	Low	22 (33.8%)
		High	30 (46.2%)
Cyclin D1 expression	Negative		8 (12.3%)
	Positive	Low	42 (64.6%)
		High	15 (23.1%)

p16 immunohistochemical evaluation

Regarding p16 expression in relation to clinicopathological parameters of the studied cases, there were statistically significant associations between p16 expression and tumor site (P = 0.005), risk stratification (P < 0.001), mitotic index (P < 0.001), local recurrence (P = 0.001), distant metastasis (P = 0.001), lymph nodes involvement (P = 0.024), and CD117 expression (P = 0.015). Also, there were statistically significant associations between P16 expression and expression of BCL2 (P = 0.05), p53 (P = 0.001), and Cyclin D1 (P = 0.018) (Table 3).

**Table 3:** Correlation of p16 immunohistochemical expression with clinicopathological parameters of the studied cases. ꭓ^2^: chi-square test; t: Student t-test; Z: Mann-Whitney U test; *: significant p ≤ 0.05. BCL2: B-cell lymphoma 2; CD34: cluster of differentiation 34; CD117: cluster of differentiation 117; DOG1: discovered on GIST-1; p16: cyclin-dependent kinase inhibitor 2A, p16INK4a; p53: tumor protein p53; S100: S100 protein; SMA:smooth muscle actin.

Clinicopathological parameters			p16		Test of significance	
			Negative (<10%)	Positive (≥10%)		
			N = 50	N = 15		
Age (years)	Mean age		61.36 ± 12.88	58.60 ± 12.19	t = 0.736	P = 0.464
Tumor size (cm)	Mean size		9.62 ± 8.01	13.77 ± 6.25	Z = 1.84	P = 0.071
Tumor site	Stomach		35 (70.0)	5 (33.3)	ꭓ^2^ = 16.57	P = 0.005*
	Small intestine		4 (8.0)	8 (53.3)		
	Rectum		7 (14.0)	2 (13.3)		
	Pelvi-abdominal		1 (2.0)	0		
	Pancreas		1 (2.0)	0		
	Mesentery		2 (4.0)	0		
Mitotic index	≤ 5 per 5 mm^2^		36 (72.0)	2 (13.3)	ꭓ^2^ = 16.35	P < 0.001*
	> 5 per 5 mm^2^		14 (28.0)	13 (86.7)		
Risk stratification	Very low/low		30 (60.0)	0	ꭓ^2^ = 20.74	P < 0.001*
	Intermediate		6 (12.0)	1 (6.7)		
	High		14 (28.0)	14 (93.3)		
Surgical cut margins	Free		47 (94.0)	13 (86.7)	ꭓ^2^ = 0.874	P = 0.325
	Infiltrative		3 (6.0)	2 (13.3)		
Local recurrence	Negative		46 (92.0)	8 (53.3)	ꭓ^2^ = 12.27	P = 0.001*
	Positive		4 (8.0)	7 (46.7)		
Metastasis	Negative		46 (92.0)	3 (20.0)	ꭓ^2^ = 32.24	P = 0.001*
	Positive		4 (8.0)	12 (80.0)		
Lymph nodes status	Negative		47 (94.0)	11 (73.3)	ꭓ^2^ = 5.13	P = 0.024*
	Positive		3 (6.0)	4 (26.7)		
CD117 expression	Negative		20 (40.0)	1 (6.7)	ꭓ^2^ = 5.86	P = 0.015*
	Positive		30 (60.0)	14 (93.3)		
DOG1 expression	Negative		26 (52.0)	5 (33.3)	ꭓ^2^ = 1.61	P = 0.204
	Positive		24 (48.0)	10 (66.7)		
CD34 expression	Negative		34 (68.0)	6 (40.0)	ꭓ^2^ = 3.82	P = 0.051
	Positive		16 (32.0)	9 (60.0)		
SMA expression	Negative		39 (78.0)	11 (73.3)	ꭓ^2^ = 0.142	P = 0.707
	Positive		11 (22.0)	4 (26.7)		
S100 expression	Negative		42 (84.0)	13 (86.7)	ꭓ^2^ = 0.063	P = 0.802
	Positive		8 (16.0)	2 (13.3)		
p53 expression	Negative		41 (82.0)	3 (20.0)	ꭓ^2^ = 20.28	P = 0.001*
	Positive		9 (18.0)	12 (80.0)		
BCL2 expression	Negative		11 (22.0)	2 (13.3)	ꭓ^2^ = 5.98	P = 0.05*
	Positive	Low	20 (40.0)	2 (13.3)		
		High	19 (38.0)	11 (73.3)		
Cyclin D1 expression	Negative		3 (6.0)	5 (33.3)	ꭓ^2^ = 8.06	P = 0.018*
	Positive	Low	35 (70.0)	7 (46.7)		
		High	12 (24.0)	3 (20.0)		

p53 immunohistochemical evaluation

Regarding p53 expression in the studied cases, we found statistically significant associations between p53 expression and tumor size (P = 0.001), tumor site (P = 0.001), risk stratification (P = 0.002), mitotic index (P < 0.001), local recurrence (P = 0.015), and distant metastasis (P = 0.001). p53 expression also showed statistically significant associations with p16 (P = 0.001), BCL2 (P = 0.003), and Cyclin D1 (P = 0.001) expressions (Table 4).

**Table 4:** Correlation of p53 immunohistochemical expression with clinicopathological parameters of the studied cases. ꭓ^2^: chi-square test; t: Student t-test; Z: Mann-Whitney U test; *: significant p ≤ 0.05. BCL2: B-cell lymphoma 2; CD34: cluster of differentiation 34; CD117: cluster of differentiation 117; DOG1: discovered on GIST-1; p16: cyclin-dependent kinase inhibitor 2A, p16INK4a; p53: tumor protein p53; S100: S100 protein; SMA: smooth muscle actin.

Clinicopathological parameters			p53		Test of significance	
			Negative (<10%)	Positive (≥10%)		
			N = 44	N = 21		
Age (years)	Mean age		60.79 ± 13.53	60.57 ± 11.0	t = 0.07	P = 0.948
Tumor size (cm)	Mean size		60.57 ± 11.02	15.36 ± 8.57	Z = 3.75	P = 0.001*
Tumor site	Stomach		31 (70.5)	9 (42.9)	ꭓ^2^ = 23.2	P = 0.001*
	Small intestine		2 (4.5)	10 (47.6)		
	Rectum		9 (20.5)	0		
	Pelvi-abdominal		0	1 (4.8)		
	Pancreas		1 (2.3)	0		
	Mesenteric		1 (2.3)	1 (4.8)		
Mitotic index	≤ 5 per 5 mm^2^		33 (75.0)	5 (23.8)	ꭓ^2^ = 15.34	P < 0.001*
	> 5 per 5 mm^2^		11 (25.0)	16 (76.2)		
Risk stratification	Very low/low		27 (61.4)	3 (14.3)	ꭓ^2^ = 12.97	P = 0.002*
	Intermediate		4 (9.1)	3 (14.3)		
	High		13 (29.5)	15 (71.4)		
Surgical cut margins	Free		41 (93.2)	19 (90.5)	ꭓ^2^ = 0.147	P = 0.702
	Infiltrative		3 (6.8)	2 (9.5)		
Local recurrence	Negative		40 (90.9)	14 (66.7)	ꭓ^2^ = 5.94	P = 0.015*
	Positive		4 (9.1)	7 (33.3)		
Metastasis	Negative		40 (90.9)	9 (42.9)	ꭓ^2^ = 17.69	P = 0.001*
	Positive		4 (9.1)	12 (57.1)		
Lymph nodes status	Negative		40 (90.9)	18 (85.7)	ꭓ^2^ = 0.399	P = 0.527
	Positive		4 (9.1)	3 (14.3)		
CD117 expression	Negative		17 (38.6)	4 (19.0)	ꭓ^2^ = 2.49	P = 0.114
	Positive		27 (61.4)	17 (81.0)		
DOG1 expression	Negative		24 (54.5)	7 (33.3)	ꭓ^2^ = 2.56	P = 0.109
	Positive		20 (45.5)	14 (66.7)		
CD34 expression	Negative		30 (68.2)	10 (47.6)	ꭓ^2^ = 2.54	P = 0.111
	Positive		14 (31.8)	11 (52.4)		
SMA expression	Negative		32 (72.7)	18 (85.7)	ꭓ^2^ = 1.35	P = 0.245
	Positive		12 (27.3)	3 (14.3)		
S100 expression	Negative		36 (81.8)	19 (90.5)	ꭓ^2^ = 0.819	P = 0.366
	Positive		8 (18.2)	2 (9.5)		
p16 expression	Negative		41 (93.2)	9 (42.9)	ꭓ^2^ = 20.28	P = 0.001*
	Positive		3 (6.8)	12 (57.1)		
BCL2 expression	Negative		12 (27.3)	1 (4.8)	ꭓ^2^ = 11.67	P = 0.003*
	Positive	Low	18 (40.90)	4 (19.0)		
		High	14 (31.8)	16 (76.2)		
Cyclin D1 expression	Negative		2 (4.5)	6 (28.6)	ꭓ^2^ = 14.39	P = 0.001*
	Positive	Low	35 (79.5)	7 (33.3)		
		High	7 (15.9)	8 (38.1)		

BCL2 immunohistochemical evaluation

There were statistically insignificant relations between BCL2 expression and all studied clinicopathological parameters except for distant metastasis (P = 0.005). In contrast, we found statistically significant relations between BCL2 expression and expressions of p16 (P = 0.05), p53 (P = 0.003), and Cyclin D1 (P = 0.048) (Table 5).

**Table 5:** Correlation of BCL2 immunohistochemical expression with clinicopathological parameters of the studied cases. ꭓ^2^: chi-square test; F: one-way ANOVA test; KW: Kruskal-Wallis test; *: significant p ≤ 0.05. BCL2: B-cell lymphoma 2; CD34: cluster of differentiation 34; CD117: cluster of differentiation 117; DOG1: discovered on GIST-1; p16: cyclin-dependent kinase inhibitor 2A, p16INK4a; p53: tumor protein p53; S100: S100 protein; SMA: smooth muscle actin.

Clinicopathological parameters			BCL 2			Test of significance	
			Negative	Positive			
			N = 13	N = 52			
				Low	High		
Age (years)	Mean age		64.46 ± 12.18	61.09 ± 13.53	58.83 ± 12.29	KW = 0.904	P = 0.410
Tumor size (cm)	Mean size		7.12 ± 4.30	10.12 ± 7.27	12.42 ± 8.89	KW = 2.24	P = 0.115
Tumor site	Stomach		11 (84.6)	16(72.7)	13(43.3)	ꭓ^2^ = 14.12	P = 0.168
	Small intestine		1 (7.7)	1(4.5)	10(33.3)		
	Rectum		1 (7.7)	3(13.6)	5(16.7)		
	Pelvi-abdominal		0	0	1(3.3)		
	Pancreas		0	1(4.5)	0		
	Mesentery		0	1(4.5)	1(3.3)		
Mitotic index	≤ 5 per 5 mm^2^		9 (69.2)	15(68.2)	14(46.7)	ꭓ^2^ = 3.19	P = 0.202
	> 5 per 5 mm^2^		4 (30.8)	7(31.8)	16(53.3)		
Risk stratification	Very low/low		8 (61.5)	11(50.0)	11(36.7)	ꭓ^2^ = 5.42	P = 0.247
	Intermediate		1 (7.7)	4(18.2)	2(6.7)		
	High		4 (30.8)	7(31.8)	17(56.7)		
Surgical cut margins	Free		12 (92.3)	20(90.9)	28(93.3)	ꭓ^2^ = 0.105	P = 0.949
	Infiltrative		1 (7.7)	2(9.1)	2(6.7)		
Local recurrence	Negative		11 (84.6)	20(90.9)	23(76.7)	ꭓ^2^ = 1.86	P = 0.395
	Positive		2 (15.4)	2(9.1)	7(23.3)		
Metastasis	Negative		12 (92.3)	20(90.9)	17(56.7)	ꭓ^2^ = 10.53	P = 0.005*
	Positive		1 (7.7)	2(9.1)	13(43.3)		
Lymph nodes status	Negative		13 (100)	20(90.9)	25(83.3)	ꭓ^2^ = 2.72	P = 0.257
	Positive		0	2(9.1)	5(16.7)		
CD117 expression	Negative		5 (38.5)	7(31.8)	9(30.0)	ꭓ^2^ = 0.301	P = 0.860
	Positive		8 (61.5)	15 (68.2)	21 (70.0)		
DOG1 expression	Negative		7 (53.8)	10 (45.5)	14 (46.7)	ꭓ^2^ = 0.254	P = 0.881
	Positive		6 (46.2)	12 (54.5)	16 (53.3)		
CD34 expression	Negative		11 (84.6)	11 (50.0)	18 (60.0)	ꭓ^2^ = 4.19	P = 0.123
	Positive		2 (15.4)	11 (50.0)	12 (40.0)		
SMA expression	Negative		9 (69.2)	16 (72.7)	25 (83.3)	ꭓ^2^ = 1.35	P = 0.510
	Positive		4 (30.8)	6 (27.3)	5 (16.7)		
S100 expression	Negative		10 (76.9)	17 (77.3)	28 (93.3)	ꭓ^2^ = 3.25	P = 0.197
	Positive		3 (23.1)	5 (22.7)	2 (6.7)		
p16 expression	Negative		11 (84.6)	20 (90.9)	19 (63.3)	ꭓ^2^ = 5.98	P = 0.050*
	Positive		2 (15.4)	2 (9.1)	11 (36.7)		
p53 expression	Negative		12 (92.3)	18 (81.8)	14 (46.7)	ꭓ^2^ = 11.67	P = 0.003*
	Positive		1 (7.7)	4 (18.2)	16 (53.3)		
Cyclin D1 expression	Negative		1 (7.7)	0	7 (23.3)	ꭓ^2^ = 9.58	P = 0.048*
	Positive	Low	11 (84.6)	16 (72.7)	15 (50.0)		
		High	1 (7.7)	6 (27.3)	8 (26.7)		

Cyclin D1 immunohistochemical evaluation

Concerning Cyclin D1 expression in relation to clinicopathological parameters, there were statistically significant correlations between Cyclin D1 expression and tumor site (P = 0.009), local recurrence (P = 0.019), distant metastasis (P = 0.024), and lymph nodes involvement (P = 0.02). Also, there were statistically significant relations between Cyclin D1 expression and expressions of p16 (P = 0.018), p53 (P = 0.001), and BCL2 (P = 0.048) (Table 6). 

**Table 6:** Correlation of Cyclin D1 immunohistochemical expression with clinicopathological parameters of the studied cases. ꭓ^2^: chi-square test; F: one-way ANOVA test; KW: Kruskal-Wallis test; *: significant p ≤ 0.05. BCL2: B-cell lymphoma 2; CD34: cluster of differentiation 34; CD117: cluster of differentiation 117; DOG1: discovered on GIST-1; p16: cyclin-dependent kinase inhibitor 2A, p16INK4a; p53: tumor protein p53; S100: S100 protein; SMA: smooth muscle actin.

Clinicopathological parameters			Cyclin D1			Test of significance	
			Negative	Positive			
			N = 8	N = 57			
				Low	High		
Age (years)	Mean age		55.63 ± 10.42	62.78 ± 12.88	57.67 ± 12.57	F = 1.67	P = 0.196
Tumor size (cm)	Mean size		12.50 ± 8.60	10.45 ± 7.37	9.93 ± 8.89	KW = 0.294	P = 0.746
Tumor site	Stomach		2 (25.0)	26 (61.9)	12 (80.0)	ꭓ^2^ = 23.42	P = 0.009*
	Small intestine		5 (62.5)	5 (11.9)	2 (13.3)		
	Rectum		0	8 (19.0)	1 (6.7)		
	Pelvi-abdominal		1 (12.5)	0	0		
	Pancreas		0	1 (2.4)	0		
	Mesenteric		0	2 (4.8)	0		
Mitotic index	≤ 5 per 5 mm^2^		3 (37.5)	28 (66.7)	7 (46.7)	ꭓ^2^ = 3.47	P = 0.176
	> 5 per 5 mm^2^		5 (62.5)	14 (33.3)	8 (53.3)		
Risk stratification	Very low/low		2 (25.0)	23 (54.8)	5 (33.3)	ꭓ^2^ = 9.05	P = 0.06
	Intermediate		0	3 (7.1)	4 (26.7)		
	High		6 (75.0)	16 (38.1)	6 (40.0)		
Surgical cut Margins	Free		7 (87.5)	39 (92.9)	14 (93.3)	ꭓ^2^ = 0.300	P = 0.860
	Infiltrative		1 (12.5)	3 (7.1)	1 (6.7)		
Local recurrence	Negative		4 (50.0)	38 (90.5)	12 (80.0)	ꭓ^2^ = 7.96	P = 0.019*
	Positive		4 (50.0)	4 (9.5)	3 (20.0)		
Metastasis	Negative		3 (37.5)	33 (78.6)	13 (86.7)	ꭓ^2^ = 7.45	P = 0.024*
	Positive		5 (62.5)	9 (21.4)	2 (13.3)		
Lymph nodes status	Negative		5 (62.5)	40 (95.2)	13 (86.7)	ꭓ^2^ = 7.63	P = 0.02*
	Positive		3 (37.5)	2 (4.8)	2 (13.3)		
CD117 expression	Negative		2 (25.0)	16 (38.1)	3 (20.0)	ꭓ^2^ = 1.88	P = 0.391
	Positive		6 (75.0)	26 (61.9)	12 (80.0)		
DOG1 expression	Negative		2 (25.0)	23 (54.8)	6 (40.0)	ꭓ^2^ = 2.85	P = 0.241
	Positive		6 (75.0)	19 (45.2)	9 (60.0)		
CD34 expression	Negative		6 (75.0)	26 (61.9)	8 (53.3)	ꭓ^2^ = 1.04	P = 0.594
	Positive		2 (25.0)	16 (38.1)	7 (46.7)		
SMA expression	Negative		7 (87.5)	31 (73.8)	12 (80.0)	ꭓ^2^ = 0.814	P = 0.666
	Positive		1 (12.5)	11 (26.2)	3 (20.0)		
S100 expression	Negative		7 (87.5)	35 (83.3)	13 (86.7)	ꭓ^2^ = 0.153	P = 0.927
	Positive		1 (12.5)	7 (16.7)	2 (13.3)		
p16 expression	Negative		3 (37.5)	35 (83.3)	12 (80.0)	ꭓ^2^ = 8.06	P = 0.018*
	Positive		5 (62.5)	7 (16.7)	3 (20.0)		
p53 expression	Negative		2 (25.0)	35 (83.3)	7 (46.7)	ꭓ^2^ = 14.39	P = 0.001*
	Positive		6 (75.0)	7 (16.7)	8 (53.3)		
BCL2 expression	Negative		1 (12.5)	11 (26.2)	1 (6.7)	ꭓ^2^ = 9.58	P = 0.048*
	Positive	Low	0	16 (38.1)	6 (40.0)		
		High	7 (87.5)	15 (35.7)	8 (53.3)		

Binary logistic regression for predictors of recurrence and metastasis

To further explore potential prognostic predictors, the relationship between the expression of the investigated immunohistochemical markers and the development of local recurrence or distant metastasis was analyzed. Binary logistic regression revealed that positive p16 expression is an independent predictor of both local recurrence (P = 0.024) and distant metastasis (P = 0.012) (Tables 7-8).

**Table 7:** Binary logistic regression for predictors of recurrence. β: beta; CI: confidence interval; *: significant p ≤ 0.05. BCL2: B-cell lymphoma 2; p16: cyclin-dependent kinase inhibitor 2A, p16INK4a; p53: tumor protein p53.

Immunohistochemical marker			β	P-value	Odds ratio	95% CI for odds ratio	
						Lower	Upper
p16 (positive)			2.790	0.024*	16.284	1.432	185.207
p53 (positive)			-0.396	0.717	0.673	0.079	5.729
BCL2	Negative		-	0.926	R	-	-
	Positive	Low	-0.589	0.696	0.555	0.029	10.637
		High	-0.412	0.768	0.662	0.043	10.265
Cyclin D1	Negative		-	0.254	R	-	-
	Positive	Low	-1.818	0.179	0.162	0.011	2.296
		High	-0.276	0.849	0.759	0.044	13.029
Constant			-2.039	0.313	0.130	-	-

**Table 8:** Binary logistic regression for predictors of metastasis. β: beta; CI: confidence interval; *: significant p ≤ 0.05. BCL2: B-cell lymphoma 2; p16: cyclin-dependent kinase inhibitor 2A, p16INK4a; p53: tumor protein p53.

Immunohistochemical marker			β	P-value	Odds ratio	95% CI for odds ratio	
						Lower	Upper
p16 (positive)			3.273	0.012*	26.401	2.083	334.550
p53 (positive)			1.282	0.264	3.604	0.380	34.169
BCL2	Negative		-	0.157	R	-	-
	Positive	Low	0.591	0.757	1.805	0.043	76.44
		High	2.749	0.139	15.629	0.410	595.07
Cyclin D1	Negative		-	0.279	R	-	-
	Positive	Low	0.005	0.998	1.005	0.024	41.994
		High	-1.889	0.331	0.151	0.003	6.846
Constant			-5.590	0.065	0.004	-	-

Survival analysis

Table 9 presents the analysis of DFS and OS, demonstrating variable prognostic implications of the investigated markers. p16 expression showed a strong association with adverse outcomes, as patients with positive expression had significantly shorter DFS (P = 0.001) and OS (P = 0.001), underscoring its value as a robust marker of poor prognosis in GISTs. Similarly, p53 positivity was correlated with unfavorable clinical behavior, with markedly reduced DFS (P = 0.001) and OS (P = 0.001), highlighting its role in identifying high-risk disease.

**Table 9:** Correlation of the studied immunohistochemical markers with disease-free survival (DFS) and overall survival (OS). ꭓ^2^: chi-square test; CI: confidence interval; DFS: disease-free survival; OS: overall survival; *: significant p ≤ 0.05. BCL2: B-cell lymphoma 2; p16: cyclin-dependent kinase inhibitor 2A, p16INK4a; p53: tumor protein p53.

Immunohistochemical marker		Median DFS (95%CI)	Log rank ꭓ^2^	P-value	Median OS (95%CI)	Log rank ꭓ^2^	P-value
p16	Negative	119.52 (109.81-129.23)	ꭓ^2^ = 36.01	P = 0.001*	123.07 (115.86-130.27)	ꭓ^2^ = 31.25	P = 0.001*
	Positive	57.27 (39.41-75.12)			77.6 (61.33-93.88)		
p53	Negative	117.82 (106.88-128.75)	ꭓ^2^ = 15.96	P = 0.001*	123.77 (117.18-130.37)	ꭓ^2^ = 16.11	P = 0.001*
	Positive	74.05 (57.07-91.02)			84 (69.05-98.94)		
BCL2	Negative	115.38 (94.05-136.72)	ꭓ^2^ = 5.32	P = 0.07	121.85 (108.34-135.35)	ꭓ^2^ = 7.16	P = 0.028*
	Low	116.72 (101.75-131.71)			123.19 (112.76-133.61)		
	High	92.23 (75.31-109.16)			100.8 (87.15-114.45)		
Cyclin D1	Negative	79.5 (57.52-101.48)	ꭓ^2^ = 4.02	P = 0.134	91.(75.98-107.02)	ꭓ^2^ = 5.11	P = 0.08
	Low	107.02 (93.22-120.83)			113.14 (102.31-123.98)		
	High	111.20 (91.94-130.46)			119.80 (106.78-132.82)		

For BCL2, patients were stratified into negative, low, and high expression groups. While DFS did not differ significantly across groups (P = 0.07), OS was significantly shorter in the high-expression group compared with the negative and low-expression groups (P = 0.028), suggesting a potential adverse prognostic impact of strong BCL2 expression.

In contrast, Cyclin D1 expression exhibited a different pattern. A stepwise increase in both DFS and OS was observed with higher expression levels. Although these differences did not achieve statistical significance for DFS or OS (P = 0.134 and P = 0.08, respectively), the pattern indicates a favorable trend associated with elevated Cyclin D1 expression.

## Discussion

GISTs constitute the most common mesenchymal neoplasms within the digestive tract. These tumors exhibit a wide range of biological behaviors. However, there are only a few histological features that help predict tumor progression. The most recent classification of GISTs considers the mitotic rate, tumor size, and tumor location, with only the mitotic rate being based on microscopic appearance. Immunohistochemistry is a helpful tool in routine pathological evaluation, facilitating more accurate diagnosis and guiding appropriate therapeutic strategies [12].

In the current study, p16 positivity was defined as strong cytoplasmic staining in ≥10% of tumor cells. We observed statistically significant associations between p16 expression and clinicopathological parameters, including tumor site, mitotic index, risk stratification, lymph node involvement, and distant metastasis. Furthermore, our findings support the role of p16 as an independent prognostic marker for local recurrence and distant metastasis in GISTs. In addition, p16 expression was significantly associated with shorter DFS and OS, highlighting its prognostic relevance.

Our findings are consistent with Schmieder et al. [13], who applied a 20% positivity threshold. They demonstrated that, in high-risk GIST patients, p16 expression was significantly linked to poor prognosis, including higher recurrence or metastasis rates and reduced OS. Moreover, p16 positivity was strongly predictive of reduced DFS in this group.

Haller et al. [14] revealed that strong cytoplasmic p16 expression was significantly associated with shorter DFS, independent of tumor location, size, and mitotic counts. Moreover, our results align with those of Jung et al. [15], who stated that p16 expression in GISTs was associated with poorer outcomes, as p16-positive tumors showed significantly higher recurrence and/or metastatic rates at both 10% and 50% cutoff values. 

In another study, Jang et al. [5] defined p16 positivity as nuclear staining in more than 20% of tumor cells, with or without concurrent cytoplasmic staining. They reported p16 expression in 92 out of 217 cases (42.4%), which was strongly correlated with higher mitotic activity, necrosis, recurrence or metastasis, and a high-risk categorization. Additionally, p16-positive GISTs were associated with significantly reduced OS and DFS compared with p16-negative tumors. Nevertheless, p16 was not identified as an independent prognostic marker.

Moreover, Yakovtsova et al. [12] defined p16 positivity as cytoplasmic staining in ≥10% of tumor cells and highlighted its significant association with aggressive behavior in GISTs. Additionally, a recent review by O’Sullivan et al. [6] reaffirmed the significance of p16 as a pivotal regulator of cell cycle progression and senescence, which biologically correlates with its prognostic importance in tumors exhibiting aggressive characteristics. This indicates that p16 overexpression signifies impaired cell cycle regulatory mechanisms, which contribute to tumor aggressiveness.

In contrast to our findings, Schneider-Stock et al. [16] reported that patients with p16-negative tumors experienced significantly worse outcomes than those with p16-positive tumors, exhibiting a 2.3-fold elevated risk of disease-related mortality. Likewise, Sabah et al. [17] established that the absence of p16 expression was frequently related to the aggressive behavior of GISTs and regarded as a negative prognostic factor. Ihle et al. [18] assessed p16 expression using an overall score derived from the mean of percentage and intensity values, with scores >2 indicating high expression. Their findings showed that p16 expression was inconsistent and did not significantly correlate with clinicopathological features or patient outcomes, suggesting the limited value of this marker in predicting aggressive behavior in GIST.

This discrepancy may be attributed to methodological variations, differences in interpretation and scoring systems, as well as heterogeneity in patient cohorts and therapeutic interventions.

In the current study, p53 positivity was defined as strong nuclear staining in ≥10% of tumor cells. A significant association was observed between p53 expression and larger tumor size, specific tumor location, higher mitotic count, local recurrence, distant metastasis, and an increased risk of disease progression. In addition, p53 expression was significantly correlated with shorter DFS and OS, emphasizing its role as a potential adverse prognostic marker in GISTs.

Our findings are consistent with those of Feakins [19], who defined p53 positivity as nuclear staining in ≥10% of tumor cells with at least moderate intensity. They demonstrated that p53 expression was more commonly detected in higher-risk GISTs, particularly those of large tumor size and increased mitotic rate. Likewise, Sabah et al. [17] found that p53 overexpression occurred more frequently in high-risk GISTs, indicating its role in tumor progression via cell cycle deregulation. Furthermore, Pauser et al. [20] considered p53 expression positive when any proportion of tumor cells exhibited strong nuclear staining. Their findings identified a notable correlation between p53 positivity and aggressive histological characteristics, such as increased tumor size, nuclear atypia, increased mitotic count, and metastatic potential.

The meta-analysis conducted by Chang et al. [21] confirmed that p53 expression was more prevalent in malignant GIST and associated with high-risk classification, thereby reinforcing its prospective role in malignancy risk stratification. Moreover, our findings align with those of Wu et al. [2], who reported in their review that p53 overexpression is significantly associated with high mitotic activity, malignancy risk, and recurrence in localized GIST.

In the study by Ihle et al. [18], p53 expression was graded using a composite score of staining extent and intensity, with values above 2 interpreted as high expression. They observed increased p53 expression with patient age and high mitotic index. Furthermore, elevated p53 expression was significantly correlated with shorter DFS. They also found that p53 overexpression was not consistently linked to TP53 mutations. In their multivariate survival analysis, no independent prognostic significance was found for any of the cell cycle regulators, including p53, could be determined.

On the other hand, Chang et al. [21] observed no significant correlation between p53 expression and tumor size or mitotic activity, indicating that p53 immunohistochemical positivity may not consistently signify malignancy across all contexts. 

Nevertheless, the accumulating evidence, particularly from recent studies, supports that p53 overexpression is frequently associated with aggressive tumor behavior and may serve as a useful prognostic biomarker in GIST.

In the current study, BCL2 expression was evaluated by combining staining intensity and percentage, and tumors were categorized as negative, low, or high expression. Our results did not show statistically significant associations with the studied clinicopathological parameters, except for distant metastasis. However, there was a significant correlation between BCL2 expression and the expressions of p16, p53, and Cyclin D1. 

Pauser et al. [20] regarded any distinct cytoplasmic staining as positive but found no correlation between BCL2 expression and prognostic indicators, thereby suggesting its limited prognostic value. In contrast, Romeo et al. [7] assessed BCL2 expression using a composite score that integrated staining intensity and the percentage of positive cells across duplicate cores, defining scores >3 as high expression. Their analysis demonstrated a significant association between elevated BCL2 expression and tumor site. In addition, Chang et al. [21] found a statistical significance between BCL2 expression and the malignancy of the GIST. Furthermore, Aoyagi et al. [22] found no significant association between BCL2 and p53 expression, indicating that these markers may operate through distinct biological mechanisms.

Regarding disease outcomes, in the current study, patients with high BCL2 expression had significantly shorter OS compared with those with negative or low expression, suggesting a possible adverse prognostic impact of strong BCL2 expression. In contrast, Kontogianni et al. [23], who evaluated 102 patients using a 10% cutoff value, reported that BCL2 expression was a favorable prognostic indicator. Similarly, Steinert et al. [24] defined BCL2 positivity as any detectable cytoplasmic staining and demonstrated that BCL2 positivity was associated with longer DFS in patients with advanced GIST treated with imatinib. These discrepancies may be attributed to differences in cutoff values, patient cohorts, and treatment modalities across studies.

In the present study, Cyclin D1 expression was evaluated using a composite score based on nuclear staining intensity and the percentage of positive cells, with tumors classified as negative, low, or high expression. Significant correlations were observed between Cyclin D1 expression and tumor site, local recurrence, distant metastasis, and lymph node involvement.

These findings are consistent with those of Kim et al. [25], who reported that Cyclin D1 overexpression defines a notably aggressive subgroup of GISTs and is substantially associated with reduced OS, even in high-grade lesions. Similarly, Sabah et al. [17] found Cyclin D1 positivity in 30.4% of malignant and recurrent GISTs, with significant correlations among other cell-cycle regulators. Draper et al. [26] further demonstrated that Cyclin D1 expression was significantly correlated with retinoblastoma protein (Rb), supporting the role of Cyclin D1/Rb pathway alterations in driving uncontrolled proliferation. Likewise, Ihle et al. [18] identified a significant correlation between higher Cyclin D1 expression and both tumor location and mitotic activity.

Beyond its clinicopathological associations, the biological and therapeutic relevance of Cyclin D1 has been highlighted in KIT-independent GISTs. Ou et al. [9] showed that high Cyclin D1 expression is indicative of imatinib resistance, while its inhibition exerts anti-proliferative effects, suggesting a potential therapeutic vulnerability. In a similar context, Chen et al. [10] demonstrated that Cyclin D1 inhibition, combined with activation of p53 and p21, reduced tumor cell proliferation, reinforcing the concept of Cyclin D1 as a possible therapeutic target in this subtype.

ِAlthough our results demonstrated significant associations between Cyclin D1 expression and adverse pathological features, patients with higher Cyclin D1 expression tended to have longer DFS and OS, albeit not statistically significant. This apparent discrepancy may be explained by the influence of therapy. Cyclin D1 overexpression may enhance tumor proliferation but, at the same time, render tumor cells more susceptible to cell cycle-targeted agents such as TKIs, thereby improving treatment responsiveness and survival outcomes. This highlights the complex and context-dependent biological role of Cyclin D1 in GISTs and emphasizes the need for further studies to clarify its dual prognostic and predictive implications.

Interestingly, in line with our survival results, Wong et al. [27] reported that Cyclin D1 overexpression might unexpectedly be associated with a more favorable prognosis, whereas Pauser et al. [20] found no significant relationship between Cyclin D1 expression and major prognostic factors such as tumor size, mitotic activity, nuclear atypia, or proliferative index.

Although many studies have explored p16, p53, BCL2, and Cyclin D1 in GISTs, consistent conclusions regarding their prognostic value remain elusive. These discrepancies may be attributed to several factors, including heterogeneity in patient populations, variations in sample size, tumor site, and risk stratification, differences in immunohistochemical protocols and scoring systems, as well as follow-up duration and treatment modalities.

Moreover, molecular and genetic differences among tumor subtypes may also influence biomarker behavior. Therefore, these variations highlight the need for further multi-centric studies with standardized methodologies, integrating molecular subtyping such as KIT, PDGFRA mutations, Succinate dehydrogenase (SDH) deficiency, and cell cycle regulatory pathways, to better define the prognostic significance of these markers.

Our study, while offering valuable insights, has limitations that should be acknowledged. These include the relatively small sample size and the retrospective design. Moreover, the study was conducted at a single center, which may limit the generalizability of the results. The immunohistochemical evaluation of biomarkers relied on semi-quantitative scoring methods, without standardized cutoff values or universally accepted scoring systems, which may introduce inter-observer variability. Additionally, some biomarker assessments were based solely on immunohistochemistry without molecular validation. These factors may affect the generalizability and interpretation of the findings.

## Conclusions

Our findings suggest that p16 and p53 expressions are valuable prognostic markers in GISTs, significantly associated with aggressive behavior and poor outcomes. p16 may serve as an independent indicator of recurrence and metastasis, while p53 could aid in risk stratification and inform therapeutic decisions. Although BCL2 and Cyclin D1 showed associations with tumor aggressiveness, their prognostic roles remain uncertain. Further multicenter and standardized studies are recommended to clarify the prognostic and therapeutic relevance of these biomarkers in GIST.
